# Adversarial Examples on XAI-Enabled DT for Smart Healthcare Systems

**DOI:** 10.3390/s24216891

**Published:** 2024-10-27

**Authors:** Niddal H. Imam

**Affiliations:** Saudi Electronic University, Prince Muhammad Ibn Salman Rd., Ar Rabi, Ryiadh 11673, Saudi Arabia; n.imam@seu.edu.sa

**Keywords:** digital twin, cybersecurity, artificial Intelligence, XAI, adversarial machine learning

## Abstract

There have recently been rapid developments in smart healthcare systems, such as precision diagnosis, smart diet management, and drug discovery. These systems require the integration of the Internet of Things (IoT) for data acquisition, Digital Twins (DT) for data representation into a digital replica and Artificial Intelligence (AI) for decision-making. DT is a digital copy or replica of physical entities (e.g., patients), one of the emerging technologies that enable the advancement of smart healthcare systems. AI and Machine Learning (ML) offer great benefits to DT-based smart healthcare systems. They also pose certain risks, including security risks, and bring up issues of fairness, trustworthiness, explainability, and interpretability. One of the challenges that still make the full adaptation of AI/ML in healthcare questionable is the explainability of AI (XAI) and interpretability of ML (IML). Although the study of the explainability and interpretability of AI/ML is now a trend, there is a lack of research on the security of XAI-enabled DT for smart healthcare systems. Existing studies limit their focus to either the security of XAI or DT. This paper provides a brief overview of the research on the security of XAI-enabled DT for smart healthcare systems. It also explores potential adversarial attacks against XAI-enabled DT for smart healthcare systems. Additionally, it proposes a framework for designing XAI-enabled DT for smart healthcare systems that are secure and trusted.

## 1. Introduction

Smart healthcare systems are the result of the fourth industrial revolution (I4.0) that shifted the world’s attention to automation and digitization [1]. Automation and data exchange are the essential goals of I4.0. They enable the development of smart factories, such as smart healthcare, via the integration of IoT, big data, AI, Cloud computing, and Digital Twins (DT). In order to design a smart healthcare system (i.e., Healthcare 4.0), self-sustaining wireless and proactive online learning systems are important [2]. An important characteristic of the first category (i.e., self-sustaining wireless) is automation and of the second category is an active data exchange and analysis. DT can play a crucial role as an enabler of the design of smart healthcare systems. Smart health can be defined as an intelligent and context-aware evolution of remote healthcare services that uses advanced technologies, such as monitoring wearable devices, mobile devices, sensors, and actuators to data, for remote support [3]. DTs can be considered to be the building blocks of the metaverse, a virtual world in which DTs interact as physical entities do in the real world [4].

DT, which are replicas of physical entities (e.g., healthcare), can be used for monitoring and testing, to suggest changes and improvements, and to support decision-making. Also, the ability to reflect, mimic and predict the status of physical systems in real time makes DT a promising technology for smart healthcare systems [5]. DT consists of three components: the real (physical) space, the virtual space and a communication medium between the two spaces [6]. Professional health carers can use DT to simulate physical objects and run experiments on the virtual copies before performing actual actions [7]. In the creation of a replica of a physical healthcare entity, the Internet of Things (IoT) can play an important role in data acquisition and the exchange of tasks. IoT enables smart machines to communicate with one another and helps build a DT of a person’s health information via wearable devices [8]. The smart healthcare system uses the Internet of Medical Things (IoMT) devices to actively monitor medical traffic. Healthcare traffic is received by an artificial intelligence (AI) enabled framework for disease prediction and remote health monitoring [9]. AI/ML algorithms can be combined with DT for data processing to make health predictions, detect early warning signs, suggest optimal treatment and personalize medicine [10]. For ML to model human DT, continuous interaction between the physical and digital spaces to obtain updated data is important [2]. In the end, AI and IoT can help DT to perform the following tasks: prediction, optimization, detection, and dynamic decision-making [1].

### 1.1. Motivations

Although as a key enabling technology for smart healthcare AI/ML has many benefits, these are offset by shortcomings such as issues with security, privacy, unfairness, and trustworthiness. The privacy of the patients’ data makes the adaptation of AI/ML for healthcare challenging. The management of smart healthcare systems relies on centralized AI/ML models that are located in the data center or cloud. This centralized architecture could lead to scalability issues [9]. It can also make AI/ML a valuable target of a security attack. Additionally, ML algorithms trained on selected biased datasets (e.g., patients with certain socioeconomic backgrounds) may fail to produce accurate results [11]. The consequences of a wrong prediction in diagnostics may cause life-changing decisions for a patient [3].

Explainability of AI (XAI) and Interpretability of ML (IML) are among the major obstacles to a full deployment of AI-enabled DT for smart healthcare systems. There is a recent trend in the use of complex ML-based models, such as deep learning models and fully-connected neural networks. Although these complex models have been proven to achieve better performances, they are often perceived as black-box models. The opposite of black-box models is white-box models which are also known as human-interpretable models. Although there is no formal consensus on the definition for explainability and interpretability of AI/ML, they can be defined as “methods and models that make the behaviors and predictions of machine learning systems understandable to humans” [12]. Interpretability and explainability are growing fields in AI/ML and this increased interest has resulted in a number of studies on different aspects of interpretable/explainable ML, from medical diagnoses to decision-making in the justice and education systems. Although the two terms interpretability and explainability are often used interchangeably in the literature, the two terms have different meanings. Interpretability enables humans to predict what is going to happen, whereas explainability enables humans to explain what is happening [13]. The ability to interpret an ML model enables decision makers (e.g., experts or non-experts) to debug, update, and ultimately trust it [14]. Additionally, ML interpretability can be either global or local. While global interpretation methods are useful for understanding the general mechanisms or debugging a model, local interpretation methods explain individual predictions [12,15].

In the context of the cybersecurity domain, there is a lack of studies that discuss the explainability and interpretability of AI/ML-enabled DT for smart healthcare systems. Existing studies focus on XAI for healthcare from the health carers’ perspective. However, several recent studies have shown that the explainability and interpretability of AI/ML can be defined on the basis of what to explain (e.g., data, features, or decisions), how to explain it when to explain it (e.g., design stage) and who to explain it to (users, health carers, or designers) [16]. Similarly, ref. [3] state that the 6W questions, Why, Who, What, Where, When, and How, need to be evaluated to design an explainable security system. The explainability and interpretability of AI/ML are very important to healthcare practitioners as they add a high level of accountability and trust and can help cybersecurity administrators debug systems. However, adversaries also can use XAI explanations to understand how the black box model works.

### 1.2. Contributions

Based on these considerations, this paper reviews the security of XAI-enabled DT for smart healthcare to answer the following research questions:

- RQ1 Do existing studies in the field of XAI-enabled DT for healthcare systems consider the robustness of the adopted XAI algorithms?
- RQ2 What does the XAI/IML of DT-based smart healthcare systems mean from the perspective of cybersecurity experts?
- RQ3 How can we improve the robustness of XAI-enabled DT for smart healthcare frameworks?
- RQ4 What are the potential adversarial attacks against XAI-based healthcare systems?

As in [17], the differences between this paper and related papers in the literature are highlighted in Table 1. It shows the coverage extent of the XAI’s security, DT’s security, Healthcare systems, and security. For example, the term “partial” refers to the level of coverage for the selected topics. To the best of the author’s knowledge, this paper is the first of its kind that attempts to explore the security of the XAI part of XAI-enabled DT for smart healthcare systems. It discussed the importance of the explainability of XAI-enabled DT healthcare systems from a cybersecurity perspective. An in-depth examination regarding the security of XAI of healthcare systems that use DT is provided. To summarize, The main contributions of the research are as follows:

1. It defines the explainability and interpretability of AI/ML-enabled DT for smart healthcare systems from a cybersecurity perspective.
2. It provides a survey of important and relevant research that discusses the security of XAI-enabled DT healthcare systems.
3. It proposes a framework of XAI/IML-enabled DT for healthcare systems.
4. It presents a simulation of potential adversarial attacks against the XAI part of a DT healthcare system.

The rest of the paper is organized as follows: Section 2 discusses related work in the field of XAI, DT and smart healthcare. A systematic literature review was presented in Section 3. Section 4 defines the explainability of AI-enabled DT for smart healthcare systems from a cybersecurity perspective. Section 5 proposes a DT framework architecture. Section 6 presents the results and analysis of a potential adversarial attack against an XAI model. Section 7 presents the limitations of the study and discusses the results of the experiments. Section 8 concludes the paper and discusses future work.

## 2. Related Work

Researchers have experimented with the creation of virtual and simulated systems (i.e., mirrored systems) of physical systems. However, the systems that were created lacked a continuous connection and real-time data exchange, which are essential elements in the “twinning” of digital to physical systems [29]. The twinning is a result of seamless interaction, communication, and synchronization between the DT, the physical twin and the surrounding environments. Authors in [22] state that mirror systems can be classified into three types: (1) a Digital Model (DM) which is an isolated system that does not have a connection to the real world; (2) a Digital Shadow (DS), which is an automatic one-way communication between physical and virtual spaces and (3) a DT which is an automatic two-way communication between the two spaces. A combination of technologies, such as big data, AI, cloud computing, and IoT allow the communication and interaction between a physical entity and its DT twin [29]. The DT concept was introduced by Michael Grieves in 2002 [30,31]. Since then, there has been an increased interest in the potential of using DT in healthcare systems. Although most studies focus on the application of DT in the context of industry [32], there is increased interest in studying the potential of DT in healthcare applications.

The Internet of Things (IoT) is an important technology for smart healthcare systems. It provides long coverage communication between different entities that can be used for data collection. Medical Internet of Things (MIoT) is a variant of IoT that has been deployed with DT in healthcare systems. Firouzi et al. [10] discuss how Wearable IoT (WIoT) has improved healthcare systems during the COVID-19 pandemic. WIoT devices enable the remote tracking of patients and the monitoring of their health for an early diagnosis. However, security and privacy issues brought up by the use of IoT are obstacles that need to be addressed. Yang et al. [33] carried out a survey on security research, threats, and open issues on MioT. Taimoor et al. [34] also discuss the challenges of the fourth-generation healthcare systems in terms of security and privacy. The authors state that although IoT and AI can improve healthcare systems, issues remain regarding their security and privacy limitations.

Another key technology that can empower DT in healthcare systems is AI/ML. The majority of AI-enabled DTs in healthcare involve human digital twins. To date, research has focused on digitally twinning some aspects of human biology [35]. It has not yet been possible to create fully functional replicas of humans. The large amount of data collected by IoT sensors needs to be analyzed in order for DT to generate a digital twin. Thus, AI/ML is considered to be the brain of DT [6]. Learning, self-correction, and reasoning are three human skills that AI tries to replicate digitally. It learns from collected data by drawing domain-specific conclusions from numerical data faster than humans can. The ability to select the best option to achieve the desired goal is an example of the digital reasoning ability of AI. Also, the third skill of AI is self-correction, which is a process of learning and repeatedly making decisions [1]. AI/ML algorithms or technologies enable DT to perform the following tasks:

- Prediction: Predicting diseases.
- Detection: Detecting early signs.
- Optimization: Monitoring disease progress.

One of the promising ML algorithms that has frequently been discussed in the healthcare field is Federated Learning (FL). Federated learning introduces a new distributed interactive AI concept for smart healthcare as it allows a number of hospitals to participate in AI training while maintaining data privacy or locally train their own model while sharing parameters with other hospitals. One of the biggest concerns of FL is that it includes data transfer mobile application usage and allows remote access to healthcare information. Also, it is vulnerable to communication cyber security attacks, such as jamming and Denial of Service a(DDoS) [25,26]. Consequently, FL improves the privacy of healthcare, but it increases the attack services. Also, the black-box nature of FL is one of the challenges that hinder adaptation in the healthcare system. Designing explainable FL could be a future research direction.

Among the biggest challenges for the full deployment of AI/ML are the explainability and interpretability of AI/ML. Helping human decision-making is one of the ultimate goals of using AI/ML. To enable this, AI/ML needs to be able to produce a detailed rationale for its decisions that can facilitate interaction with humans. This is known as AI explainability. Kobayashi et al. [5] state that XAI is important for accurate predictions. The authors discuss the importance of complex ML for DT-enabled systems in prediction and system update. They define explainability as allowing users to understand the most important factors in the model’s prediction and interpretability as ensuring that the model predictions can be understood by non-technical users. Authors in [36] studied the importance of XAI and IML for AI applications designed for healthcare and medical diagnosis. They conclude that using existing libraries for the model’s interpretability with XAI frameworks and other clinical factors helps humans understand “black box” AI.

The security and privacy of AI/ML are among the challenges that make health carers hesitant about fully adopting AI/ML-enabled systems. The robustness of ML-based models against adversarial attacks has recently become subject to increased interest in the research community [37]. Although ML-based models have been widely used to automate different types of systems, these models are vulnerable to well-crafted, small perturbations. The vulnerability of ML has been examined by a large number of studies, where authors develop frameworks for evaluating algorithms [38,39], launching attacks against ML models, and designing countermeasures [40]. Also, authors in [38,39,41] propose frameworks for evaluating the security of ML and envisioning different attack scenarios against ML algorithms. The framework suggests the following steps: (1) identify potential attacks against ML models by using the popular taxonomy; (2) simulate these attacks to evaluate the resilience of ML models and assume that the adversary’s attacks are implemented according to their goals, knowledge, and capabilities/resources and (3) investigate some possible defense strategies against these attacks. Defending against adversarial attacks is challenging because these attacks are non-intrusive in nature. Thus, designing proactive models rather than traditional reactive models is a necessity for AI/ML-enabled healthcare systems. This has motivated researchers to formulate different attack scenarios against machine learning algorithms and classification models and propose some countermeasures [42].

Designing XAI-enabled systems is challenging because interpretability and accuracy are two competing concepts. Senevirathna et al. [3] add that explainability is regarded as a third property constraining performance and security. Simplification and generalization are the main concerns of interpretability, as accuracy favors nuance and exception [14]. There are two approaches that are widely used for interpretations of ML models: regression analysis and rule-based ML [12]. These models provide descriptions of a class as well as predicitions [43]. On the one hand, linear regression models can be interpreted by analyzing the model structure or a weighted sum of features. For example, the weights can be interpreted as the effects that the features have on the prediction. On the other hand, rule-based ML models, such as decision trees [44] or decision sets [43], interpret a learned structure (e.g., IF-THEN) to understand how the model makes predictions. Some recent studies have attempted to make complex models interpretable; for example, refs. [36,45] have visualized the features of CNN and [46] the important features of a random forest. However, in high-dimensional scenarios, linear regression, decision trees, or complex models may become not interpretable [12]. Additionally, a decision-set type of rule-based ML has some shortcomings. Understanding all of the possible conditions that must be satisfied is difficult and limits interpretability. This is especially so in multi-class classification [14] or complex AI/ML-enabled healthcare systems [15].

Although, increasingly, there are methods being developed that explain how algorithms work and reach their final decision (Designing AI Using a Human-Centered), there is a lack of studies that focus on designing methods for the security of AI explainability. To design an explainable AI-based system, the researcher needs to answer the following questions: What to explain? Who to explain to? How to explain? and Why to explain? The answer to these questions regarding XAI for cybersecurity will be different according to the target audience [16]. Some studies suggest overcoming the XAI shortcomings by removing humans from the loop, which is dangerous in the healthcare domain. Others propose using less complex AI models, which may lead to systems that do not provide the desirable results. Thus, the degree of explainability is another important question to ask. Taimoor et al. [34] state that XAI research includes feature engineering, developing and testing algorithms, risk and opportunities, ethical considerations, and trust. We believe that robustness to adversarial examples needs to be included. Authors in [47] state that IoT and AI are not secure solutions. The number of publications that survey DT in terms of definitions, characteristics, development, and application has increased, but there is a lack of studies that focus on the security of DT-based healthcare systems. Designing a simplified approximation model to make complex models interpretable to cybersecurity administrators/experts is one of the goals of this paper. The level of AI explainability/ML interpretability to users should be evaluated as it may make the system vulnerable to adversarial attacks. Thus, this paper focuses on exploring the security of AI explainability as part of smart healthcare systems that use DT.

## 3. A Survey of Related Works

This section aims to answer the following question: (RQ1) Do existing studies in the field of XAI-enabled DT for healthcare systems consider the robustness of the adopted XAI algorithms? A systematic literature review has been conducted by identifying the top research findings in the domain of the security of XAI-enabled DT smart healthcare systems. The following subsections describe the methodology followed for extracting articles, selection criteria and filtering processes.

### 3.1. Methodology

The current literature was summarized and analyzed by following the methodology introduced in [48,49]. This SLR was conducted in three stages: (1) defining the search keywords, (2) selecting the list of databases to be used for the search, and (3) defining the inclusion and exclusion criteria.

#### 3.1.1. Procedures for Defining Keywords and Data Sources

Following the procedure proposed in [50], the research questions were used to define a list of keywords to be used for search queries. The following is the defined search query: “(Explainable AI OR XAI) AND (Interpretable ML OR IML) AND Healthcare AND (“Digital Twin” OR DT) AND security”. Then, related research papers were downloaded using the selected string of keywords. Google Scholar was selected as our data source. As suggested by [29], using Google Scholar as the main web search engine helps avoid bias towards any specific publishers. Only English papers published between 1 January 2013 and 1 December 2023 were included.

#### 3.1.2. Eligibility Criteria

**Figure 1:**
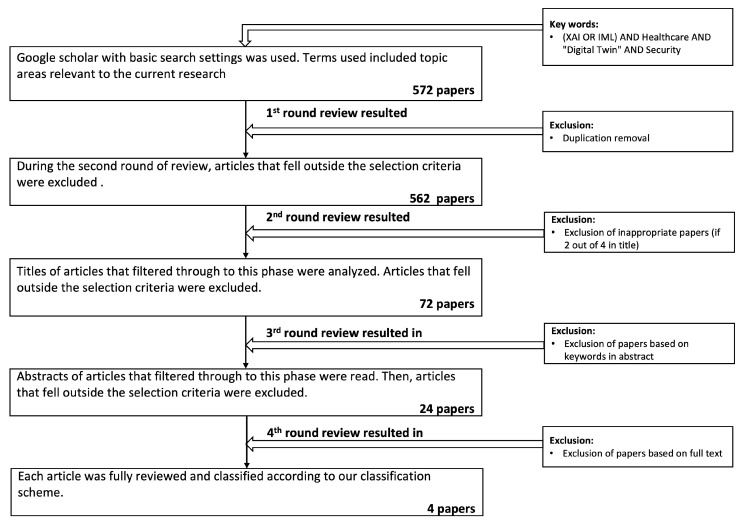
Number of research papers identified and reasons for exclusion.

The inclusion–exclusion criteria were applied at five levels (see Figure 1), and ineligible studies were eliminated after each level. A list of inclusion criteria (IC) and exclusion criteria (EC) were defined and applied as follows:

- IC 1: A well-discussed studies that report at least two out of the keywords.
- IC 2: Articles written in English language.
- IC 3: Articles published in a peer-reviewed journal or a conference.
- IC 4: Articles published in the last ten years were included (i.e., 2013–2023)
- IC 5: Relevance to the security of XAI-enabled DT for smart healthcare systems
- EC 1: Thesis, news articles, reports, or websites were excluded.
- EC 2: Articles published in languages other than English were excluded.

Any articles published in a language other than English were excluded. Only articles published in the last decade were included (i.e., 2013–2023). Theses, news articles, reports, or websites were excluded.

#### 3.1.3. Results

We identified 572 articles from different publishers, including MEDLINE, Web of Science, IEEE Xplore digital library, and ACM digital library. After removing 10 duplicated articles, the titles of 562 articles were screened. Articles with titles that did not include at least two out of the four keywords (XAI, DT, healthcare, and security) were excluded. The abstracts of the remaining 72 studies were then screened based on the keywords. Twenty four articles were retained for a full-text review, resulting in only four articles being deemed relevant to include in the final full-text extraction. Figure 1 depicts the SLR’s flow chart.

Table 2 presents a comparative analysis of the existing work on the security of XAI-enabled DT for smart healthcare systems. The analysis of 14 articles is provided in the table. Four topics were considered for the comparisons. Topics (XAI, DT, Healthcare, and security). In the table, four colors (High coverage, medium coverage, low coverage, or not applicable) were used to show the extent of coverage on the four topics for each article. Only 4 out of the 562 articles identified were found to focus on the security of XAI-enabled DT for smart healthcare systems. Although 24 articles were identified in a full-text review, 14 articles were selected for the comparative analysis as they discussed some of the security challenges of smart healthcare systems. The results show that only four articles were found to partially focus on the security of the adopted XAI algorithms of DT-based smart healthcare systems. This shows that more research in this area is needed. Following are summaries of the four papers that were included in the final full-text extraction.

In [18], the authors propose a framework for the healthcare metaverse that contains three environments: (1) the healthcare practitioners’ environment, (2) the metaverse or virtual environment, and (3) the patient environment. The framework includes different technologies (i.e., XAI, Blockchain, DT) to provide virtual health services. Doctors need to enter the doctor’s environment from which they can interact with patients. Patient treatment requests are processed in the patient environment by nurses, caregivers, and robots. In the metaverse environment, which is the main part of the framework, avatars of patients, medical staff, and doctors can interact with one another. The XAI is used to provide logical reasoning for the framework’s predictions, and the blockchain was incorporated to ensure patients’ data privacy and track users’ activities. Although the authors discuss data privacy and security concerns and include the blockchain to address them, the vulnerability of XAI to adversarial examples was not considered.

In [5], the authors investigate the importance of XAI and IML in the prognostic and health management (PHM) that uses a Digital Twin framework. The remaining useful life (RUL) was used as the explanation parameter target. The authors employ different XAI and IML tools/methods to study the target model. They conclude their study by observing that the prediction accuracy of RUL is affected by the adopted explainable or interpretable AI. The paper presents a detailed investigation of the importance of XAI and IML for a DT-based healthcare framework but does not consider the presence of adversaries who may abuse the explainability and interpretability of the target framework.

An investigation of using AI for improving the security of DT-based systems was performed in [22]. The authors discuss possible cybersecurity attacks, such as MITM, and DDoS, on DT’s network and IoT. As a potential countermeasure for such threats, the authors state that AI can play an important role in improving the robustness of IoT and DT platforms. For example, FL can ensure the exchange of data/information privately, and the explainability of XAI can help with the detection of adversarial examples. On the other hand, there are methods that use AI to attack DT, such as infiltration, poisoning, or exploratory attacks. The paper focuses on the security of DT but does not investigate the security of XAI when used as part of a DT-based healthcare system.

The authors of [17] investigate how XAI can improve the security and privacy of eHealth data. Security properties to protect data were defined as follows: anonymity, accountability, authenticity, confidentiality, integrity, non-repudiation, and revocability. The authors discuss existing and potential approaches to data privacy, such as cloud computing, blockchain, and encryption. Briefly, they discuss the importance of XAI tools in improving security and privacy through global or local explainability. However, the security of XAI when used in eHealth was not discussed.

## 4. XAI from Cybersecurity Perspectives

One of the research questions that this paper is trying to answer is (RQ2) what does the XAI/IML of DT-based smart healthcare systems mean from the perspective of cybersecurity experts? To answer this question, two points are considered: (1) whether there is any difference between AI’s explainability and interpretability and (2) why it is important to define whether the XAI/IML is designed for security purposes. First, the explainability and interpretability of AI/ML are always used interchangeably, but some recent studies show that these are not the same. Thus, the first step to understanding the XAI/IML of DT-based smart healthcare systems from a cybersecurity perspective, explainability and interpretability need to be defined clearly. Kobayashi et al. [5] define XAI as the ability of an AI system to provide reasoning behind its actions (i.e., predictions and decisions), whereas IML is the process of explaining the relationship between a model’s input and output variables to the decision-making process. Also, Fischer et al. [13] state that interpretability enables humans to predict what is going to happen, but explainability enables humans to explain what is happening. This shows that it is very important for cybersecurity administrators/analysts to distinguish between the two as interpretability (a.k.a pre-model explainability) is crucial at the design stage and explainability is crucial after an attack and at the debugging stage. However, it may not be important for doctors or nurses to learn about the correlation between input and output samples to the prediction process; they may be more interested in the reasoning behind the predictions.

The second important point to be taken into consideration when addressing this research question is that one of the two categories of adversarial attacks against AI/ML is an exploratory attack, where an adversary tries to learn some of the characteristics of AI/ML. In the case of XAI/IML, adversaries may take advantage of the explainability and interpretability of an XAI/IML. In other words, as the adversaries’ knowledge increases, the effectiveness of the attacks also increases. This idea supports the statement discussed in [3] about the importance of defining the XAI/IML’s involved to improve accountability. The authors state that by answering the 6W questions, Why, Who, What, Where, When, and How explanations would be generated. Table 3, inspired by [3,51], shows the steps for designing XAI. For smart healthcare systems, stakeholders can be categorized into the following groups: creators (i.e., developers, testers, or cybersecurity experts), system operators who manage systems on a daily basis, and end users (i.e., doctors or nurses). The granularity of explanations should be different for these groups. For instance, system operators need a level of explanation that is higher than the explanation needed by end users and slightly lower than the explanation needed by the creators. Another thing that necessitates the identification of XAI stakeholders is that XAI does not support solely the decision making, but also the security and accountability. Thus, the purpose of adapting XAI is different for stakeholders.

To summarize, the requirements of designing XAI/IML-enabled DT for smart healthcare systems from the cybersecurity experts’ perspective are different as the objective, granularity, and methods used for explanation are different. Also, the expertise level required is different and thus requires distinct explanations [52]. Answering the 6W questions is important; existing literature often discusses XAI, in general, without differentiating the stakeholders. For example, ref. [53] state that visual-based XAI methods could be useful for users with low AI/ML domain knowledge; whereas, some of the existing XAI tools or libraries require a significant amount of technical knowledge.

## 5. A Secure DT Architecture

The results of the existing studies show that the security of XAI algorithms in DT-based healthcare systems is overlooked. This section aims to answer (RQ3) by reviewing existing XAI-enabled DT frameworks and adding a new security layer.

DTs create a digital replica of a physical object using IoT, AI, and communication technology. Cybersecurity issues of DT have not yet been sufficiently explored which is a problem because DTs are a critical part of the automation process and represent the digital copy of the physical world. Also, because DTs consist of three spaces, the physical, communication, and digital space, potential adversarial attacks against them vary. For instance, adversaries can increase computational overheads to corrupt the processes of generating replicas, manipulate required information of the representation models, or take control of physical objects from the digital space [54]. Thus, when DTs are used in critical systems (i.e., healthcare), considering the vulnerabilities of DTs becomes a must. Since the XAI is the brain of DTs, the vulnerability of the XAI will be examined in the following section.

### Proposed DTs Architecture

There are different approaches for designing DT’s architecture layer models in the literature. Reference [22] stated that existing frameworks have some limitations. For example, the functional details required for building a functional architecture are missing, and some models cannot be generalized. To design a secure XAI-enabled DT framework, the conceptualization of a DT’s layers needs to be considered. Frameworks presented in [22,54,55] were adopted and updated. The proposed DT framework consists of five layers that are described below:

- Layer 1—data collection and acquisition: technologies (i.e., cyber-physical systems (CPS) and IoT (IIoT)) are used to capture the dynamics of the physical space and prepare the control instructions for the physical assets
- Layer 2—data management and analysis: Big Data (BD) and AI/ML are needed for data management, analysis and decision-making. Cloud, fog, and edge are the best computing infrastructures for processing and analyzing big data. These allow Layer 3 services to be executed.
- Layer 3—data modeling and additional services: XAI tools can be used to provide reasoning for the Layer 2 decisions and predictions. Also, tools, such as CAD/ECAD electronic computer-aided) systems and CAM (computer-aided manufacturing) have been used to characterize states, behaviors and shapes of a physical object. Also, it provides additional services, such as recommendations, encryption, and cybersecurity detection.
- Layer 4—model evaluation and verification: Human-in-the-loop approach is integrated to evaluate the security of the designed model before the virtualization.
- Layer 5—data visualization and accessibility: Allows end users to visualize digital models to make decisions regarding physical objects.

**Figure 2:**
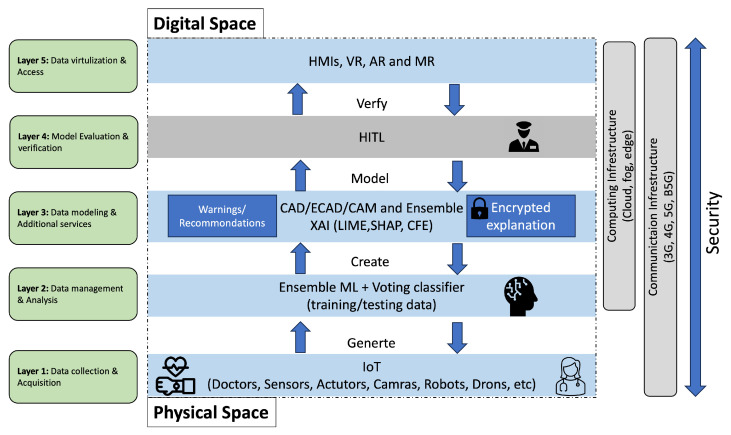
Proposed DTs framework and layer.

Figure 2 depicts the five layers of the Digital Twin framework. The figure is adapted from [22,35,54,55] and updated. Physical space data are collected by Layer 1 via wearable devices, cameras, and medical records. Collected data are managed and processed in Layer 2 using AI/ML algorithms to prepare data for Layer 3. An ensemble of ML algorithms with a voting classifier can be used for capturing different features, improving accuracy, and detecting errors. In Layer 3, XAI has been added as an additional service along with data modeling technologies, such as ECAD, and CAM. The explanation of the created model is important for healthcare practitioners and cybersecurity experts for the reasons that have been discussed above. Reference [5] states that understanding the explainability of the AI/ML algorithms used in the DTs framework from the decision-making aspect is crucial. Also, XAI is important for updating DT frameworks, which is a key component that is not considered in ordinary simulations. Thus, an ensemble of XAI tools can be employed in Layer 3 to provide different levels of explanations (i.e., global and local), which can be used to generate warnings if there is a disagreement. Layer 3 not only models the collected data, but it provides the reasoning behind its decisions using the XAI tools and warnings based on the generated explanations. A new layer was added between Layer 3 which produces a model of the physical space and the virtualization in Layer 5. Evaluating the generated warnings or recommendations from Layer 3 requires a certain amount of human work, although this may be costly and time-consuming [56]. Also, ref. [57] states that for some practical tasks (e.g., medical diagnosis) humans need to verify labeled data. Hence, the integration of the human-in-the-loop (HITL) approach in the DT’s architecture is crucial. To the best of the author’s knowledge, this is the first study that integrates XAI and HITL into the DTs architecture and proposes a security layer for verification.

Figure 2 shows that the data flows in both directions (upward and downward) of the five layers to enable the deployed XAI to update both the physical and digital spaces. The connectivity between the physical and digital spaces of DTs is a major concern; if they were absent, the DTs would be useless [58]. Thus, B5G or 6G can be used to facilitate the communication between the DTs’ components, and cloud, fog, and edge are used for computation. The integration of these advanced technologies and computation systems presents serious security concerns at all layers. Reference [54] adds that any security analysis of DTs needs to consider the functionality layers in terms of availability, integrity and confidentiality. The authors categorize attack surfaces into digital and physical. The first includes software and components that provide resources for computation; whereas, the physical attack surface comprises all security threats associated with CPS/IIoT nodes, communication infrastructures and facilities. Since the attack surface is very wide, potential adversarial attacks against the XAI part of the DTs were simulated as described in the following section.

## 6. Adversarial Attacks Against XAI

The aim of this section is to answer (RQ4) What are the potential adversarial attacks against XAI-enabled DT for healthcare systems? Despite the fact that XAI can play an important role in improving the trustworthiness, transparency, and security of DT-based healthcare systems, their explainability can also be used to compromise the system. It is important to consider vulnerability to cyber attacks for both the AI models deployed and the explainability part [59]. Employing XAI increases the attack surface against smart healthcare. Falsifying the explainability can be a target for an attacker [3]. Adversaries can modify explanations (i.e., post-hoc) without affecting the model’s prediction which may cause a stakeholder to make the wrong decision [3]. Thus, designing proactive XAI-enabled DT for healthcare systems rather than traditional reactive systems is a necessity in an adversarial environment, where the arms race between system designers and adversaries is never-ending. Since reacting to detected attacks will never prevent future attacks, proactively anticipating adversaries’ activities enables the development of suitable defense methods before an attack occurs [39]. This has motivated us to formulate an attack scenario against the XAI part of DT-based healthcare systems using existing proposed frameworks [15,38,39,60].

A taxonomy proposed in [38,39,61] was adapted and updated to simulate a potential attack scenario against XAI algorithms considering the four following axes:

The attack INFLUENCE
  - **Causative:**
    The attack influences the training data to cause wrong predictions (i.e., poisoning attacks).
  - **Exploratory**: The attack exploits knowledge about the deployed classifier to cause wrong predictions without influencing training data (i.e., evasion and privacy attacks.).
The type of SECURITY VIOLATION
  - **Integrity violation**: An adversary accessing algorithm’s explanation without compromising normal system operations.
  - **Availability violation**: An adversary compromises the normal system functionalities available to legitimate users.
  - **Privacy violation**: An adversary obtains private information about the system, such as algorithms’ explanations.
The attack TARGET
  - **I-attacks** target the explanation of the XAI model.
  - **CI-attacks** target the explanation and prediction of the XAI model.
The attack SPECIFICITY
  - **Targeted** attacks focus on a particular instance.
  - **Indiscriminate** attacks encompass a wide range of instances.

The first axis, which is the attack influence, concerns an adversary’s capability to influence an XAI’s predictions/explanations. The influence is causative if an adversary misleads the deployed XAI by contaminating (poisoning) the training dataset via injecting carefully crafted samples (a.k.a adversarial example) into it. On the other hand, the influence is exploratory if an adversary gains knowledge about the deployed XAI to cause mis-explanation at the testing phase without influencing training data.

The second axis describes the type of security violation committed by an adversary. The security violation can be regarded as an integrity violation if it enables an adversary to alter an XAI model’s explanation. Also, the attack can violate the XAI model’s availability if it creates a denial of service, where it prevents legitimate users from accessing the system’s explanation. Additionally, the security violation can be regarded as a privacy violation if it allows an adversary to have authorized access to the XAI’s explanation.

The third axis, which was adapted from [62], specifies the attackers’ target. Attackers can either target the explainability or prediction of the XAI model or both. In ***I-attacks***, attackers attempt to attack the single explanation without affecting the prediction of a deployed classifier. In the ***CI-attacks***, attackers attempt to concurrently compromise the integrity of the classifier and explanation.

The fourth axis of the taxonomy refers to the specificity of an attack. It indicates how specific an adversary’s goal is. The attack specificity can be either targeted or indiscriminate, depending on whether the attack (1) causes the XAI model to misclassify a single or few instances, or (2) undermines the model’s performance on a larger set of instances.

The existing literature on adversarial ML models provides different attack examples and defense methods for both adversarial attack types (causative and exploratory). Ref. [15] presents a taxonomy of potential attacks against ML models (see Table 4).

### 6.1. Threat Models

Threat modeling, which involves defining an adversary’s goal, knowledge, and capability [38,39,61], is an important step toward identifying potential attack scenarios. The attacker’s goal can be based on the type of security violation, on the attack target, and on the attack specificity. For instance, the adversary’s goal could be to violate the XAI models’ integrity by manipulating a specific instance to cause a wrong explanation. An attacker’s level of knowledge about the XAI models varies and may include perfect knowledge (having full access to XAI’s explanation), limited knowledge (having limited access to XAI’s explanation), or zero knowledge (not having access to XAI’s explanation). An attacker’s capability can enable them to either influence training data (causative attack) or testing data (exploratory attack).

### 6.2. A Potential Attack Scenario

Here, an experiment that depicts a possible scenario of an adversarial attack against the XAI part of a DT-enabled healthcare system is discussed. One of the most common types of a causative attack is a poisoning attack, in which an adversary contaminates the training dataset to affect ML models’ output [38]. A label-flipping attack, which is an example of a causative attack, was chosen for the experiment. In a label-flipping attack, an adversary flips the label of some samples and then injects them into the training data. Different methods have been used to perform this attack in the literature and, the easiest method is to randomly flip the label of some samples that may be used for retraining. In [63], it was shown that randomly flipping about 40% of the training data’s labels decreased the prediction accuracy of the deployed classifier. However, as the experiment in this current paper focuses on the XAI model, the model’s explanation was used to select samples to be flipped.

The settings of the attack scenario are as follows: *the adversary’s goal* is to violate the integrity and privacy of the XAI model, the attack target is ***CI***, and the attack specificity can be either targeted or indiscriminate. In *The adversary’s capability*, it is assumed that the adversary is capable of influencing the training data. *The adversary’s knowledge* is assumed to be perfect (white-box setting). Within such a scenario, a potential attack strategy is as follows:

- Adversaries can use the model’s explanation that either has been inferred through probing or gained via unauthorized access (privacy violation).
- Depending on the knowledge that the adversary gains, they can select samples that need to be flipped in the training dataset.

### 6.3. Experimental Settings

This section presents and discusses the experimental results and evaluation. Experiments were run on Linux Ubuntu 18.04 LTS operating system with an Intel(R) Core(TM) i7-8750H CPU 2.20 GHz x 12 of 983.4 GB memory. A model built in (https://www.kaggle.com/code/joshuaswords/predicting-a-stroke-shap-lime-explainer-eli5/notebook (accessed on 6 April 2024)) was used for implementation and a Stroke Prediction Dataset (https://www.kaggle.com/datasets/fedesoriano/stroke-prediction-dataset (accessed on 6 April 2024)) was used. The dataset contains 5110 unique IDs for patients with 12 attributes, such as gender, age, disease, and smoking status. Each row in the dataset, which provides relevant information about the patient, is classified into two classes: 1 if the patient had had a stroke or 0 if not. Different ML algorithms were used for the experiments in this section.

#### 6.3.1. Evaluation Metrics

The following evaluation metrics have been used to measure the models’ performance: accuracy, recall, precision, and F1 score. These metrics, along with their descriptions, are defined in Table 5 [15,64,65].

#### 6.3.2. Models’ Design

The experiments conducted in this section focused on using the adopted models to predict whether a patient is likely to suffer a stroke based on 11 clinical features. The same settings used by the author of the adapted models were followed. First, the dataset was processed to find missing values and explore the features. Then, the dataset was split into training and testing for model preparation. Also, the SMOTE (Synthetic Minority Over-sampling Technique) was used to balance the dataset. Three algorithms Random Forest (RF), Support Vector Machines (SVM), and Logistic Regression (LR) were selected for the classification task. After building the models, a 10-fold cross-validation was used for evaluation. The results in Table 6, Table 7 and Table 8 show that Random Forest performed the best with 88 accuracy.

#### 6.3.3. Models’ Explainability

**Figure 3:**
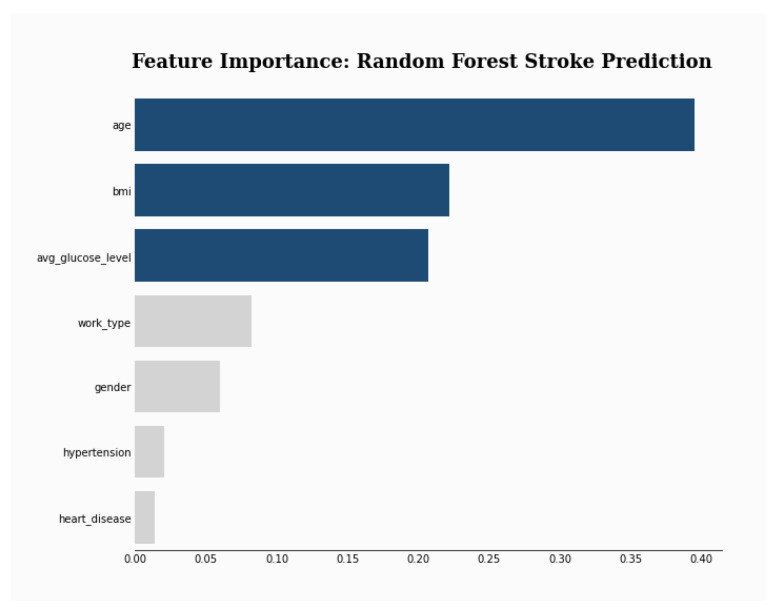
Feature Importance.

To explain the results of the selected model (i.e., RF), a feature selection and three explanation methods were used. First, the RF feature importance was plotted and the result shows that age is by far the most used feature by the model (see Figure 3).

**Figure 4:**
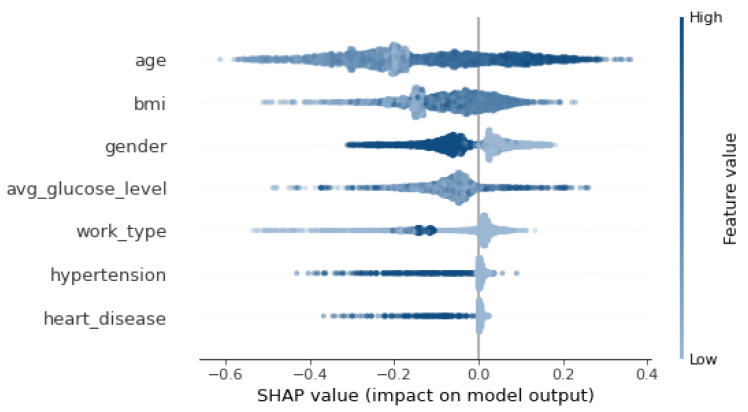
SHAP Explanation.

Then, three popular XAI frameworks, namely, SHAP [66], LIME [67], ELI5 [68] were used. SHAP (SHapley Additive exPlanations) is a tool for determining the contribution of each feature to the model’s prediction. LIME (Local Interpretable Model-agnostic Explanations) was used to interpret the prediction of the model on a single instance. ELI5 stands for “explain like I am 5” and aims to explain the prediction of any model. The results shown in Figure 4, Table 9 and Table 10 show that SHAP and ELIS (i.e., global explainers) rank age and then bmi as the most important features. On the other hand, LIME, which is a local explainer, ranks gender and bmi as the most important features.

#### 6.3.4. A Simulation of Potential Adversarial Attack

To simulate a label flipping attack as discussed in Section 6.3, the labeled 10% of the dataset was flipped considering the most important feature (i.e., age); 25 out of 250 labels, which account for 10% of label 1 were flipped. Labels of patients aged 2 were flipped to 1, thus labeling 2-year-old patients as being at risk of suffering a stroke. The performance of the three ML models was evaluated using the contaminated dataset. The results in Table 11, Table 12 and Table 13 show that the performance of the SVM and LR was slightly degraded, but it remained almost the same for RF. However, the results of the four explanation methods showed that the label-flipping attack affected the explanation. Although the literature suggests that flipping about 40% of the training data labels decreased the accuracy, 10% of the training data decreased the performance of the adopted model in this paper.

**Figure 5:**
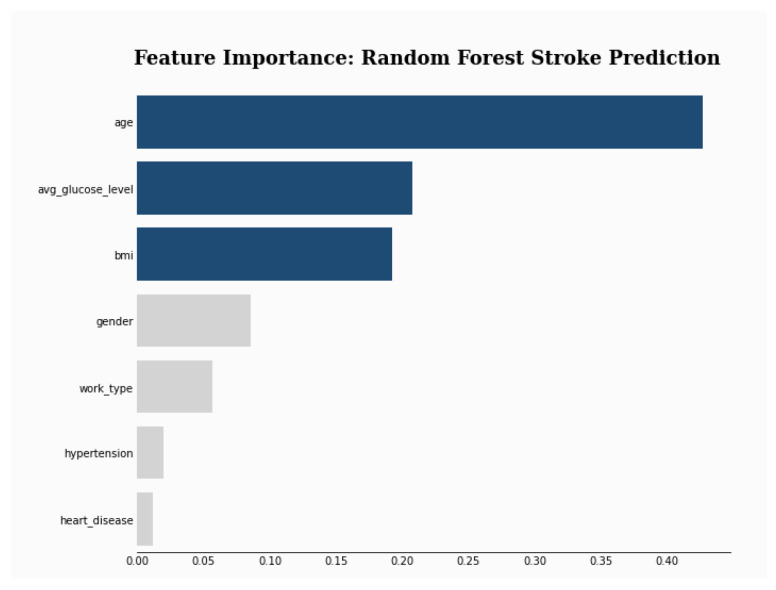
RF Feature Importance After the Label-flipping Attack.

**Figure 6:**
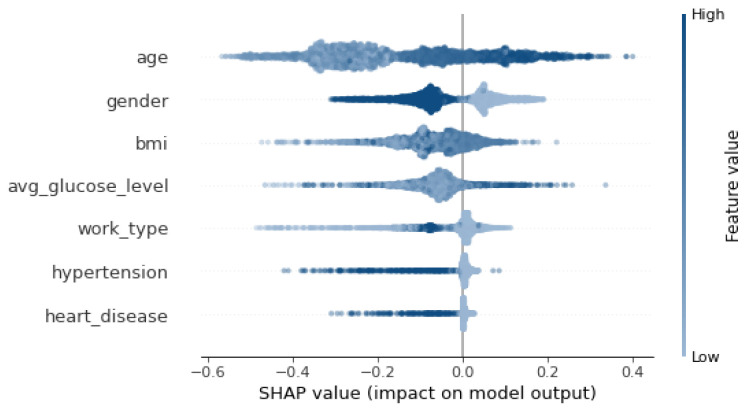
SHAP Explanation After the Label-flipping Attack.

Figure 5 shows that feature importance order has changed as BMI is replaced by avg_glucose_level. Also, Figure 6 depicts an order change in the feature importance for the SHAP explainer. Gender becomes the second most important feature instead of BMI. LIME explainer Table 14 ranks gender and work_type as the most important features, which is different from the original ranking. Moreover, work_type replaces BMI in the ELI5 explanation Table 15.

## 7. Discussion

One of the challenges in designing an adversary-aware XAI is that explainability is much more difficult to measure than accuracy or performance as the level of an explanation is subjective [69]. Also, ref. [59] adds that the XAI output is as crucial as the format of the explanation output and could have a strong influence on certain users. For example, a text-based explanation is commonly used in the Natural Language Processing (NLP) field, but visualized explanation approaches are used in healthcare. Also, the explainability of the deployed model can reveal valuable information to adversaries. To attack ML-based models that do not provide an explanation, adversaries need to probe the model to collect information. However, the XAI produces helpful information for adversaries. One of the solutions proposed in the literature is to provide stakeholders with the minimum level of explanation that enables them to achieve their goals. Model explanations should be categorized according to the stakeholders’ privileges. The models’ explanations (i.e., pre-model and in-model) need to be encrypted and the model should not reveal detailed information about features and algorithms. It can provide users with a warning message that consists of a prediction description, and an explanation of the hazards and consequences (i.e., post-model) [62]. However, users with higher privileges can query the model to access the encrypted detailed explanation. Another defensive method is encrypting the explanation, so that only stakeholders with a high level of privileges can access it. Moreover, delaying the explanation availability can disrupt the adversaries’ attack process [3].

The proposed human-in-the-loop approach layer between layer 3 and layer 5 is needed for verification. The explanation can be used by the system administrators to detect adversarial examples (i.e., explanation-based security approach). For example, if the deployed XAI predicts that a patient has high blood pressure, the explanation (i.e., feature importance) needs to be evaluated. Also, using the ensemble of XAI tools for adversarial example detection can help with the verification task before layer 5 by alerting administrators in case of an explanation disagreement. For this solution to work, systems designers and medical professionals need to design explanation messages based on the XAI features. There are some features that indicate certain types of disease. Consequently, a sudden change in such features is regarded as a sign of an adversarial attack or a disagreement on feature importance and the explanation message is a sign of an adversarial attack against XAI’s explanation.

Grey-box and white-box attacks, such as the label-flipping attack presented earlier, can be successful against XAI. Designing an adversary-aware XAI-based DT healthcare system considering the adaptability of the deployed model is important as an increase in the false positives and false negatives rate can lead to life-threatening consequences. Also, “the life-critical nature of healthcare applications demands that the developed ML systems should be safe and robust and should remain robust over time” [70]. Consequently, the DT framework proposed in this current paper uses an ensemble of XAI tools to help detect adversarial examples by considering the disagreement between the deployed classifiers and using the detected adversarial examples to update the framework (i.e., adaptability). Another important point that needs to be considered at an early stage of designing an XAI DT-enabled healthcare system is involving humans in data labeling as data generated by artifacts (i.e., IoT, cameras, robots) lacks context. Manual data labeling is about adding extra information or metadata to a piece of data [16]. Metadata increase the explainability of AI and helps with feature engineering, detecting incorrect labels, and detecting adversarial examples.

Additionally, one of the promising solutions for protecting privacy in smart-health systems, such as XAI-enable DT, is federated learning (FL) [71]. It enables models to be trained in a distributed training fashion by averaging the local model updates from multiple IoMT networks without accessing the local data [9]. As a result, potential risks of gaining unauthorized access to the dataset used to train models can be mitigated. However, this will not protect the likelihood of the explanation disclosure.

## 8. Conclusions

This paper provided a detailed overview of research on the security of XAI-enabled DT for smart healthcare systems. Also, it defined the explainability of AI-enabled DT for smart healthcare systems from a cybersecurity perspective. An in-depth examination of a potential adversarial attack against the XAI part of DT healthcare systems was also presented. A model designed to predict the likelihood of a patient suffering a stroke was adapted and used as a case study. A label-flip attack was launched against three adapted models that use four explanation methods. The results showed that a label-flipping attack could affect the models’ explanation. An adversary-aware DT framework for smart healthcare was also proposed in this paper.

There are some limitations that future research needs to consider. In this paper, the adopted model that has been evaluated against a label-flipping attack uses traditional ML algorithms. Future research may use more complex or cutting-edge healthcare models to be evaluated against adversarial attacks. Also, future research should focus on evaluating the robustness of different XAI tools against other adversarial attacks; simulating more attacks against XAI-enabled DT systems and designing an adversary-aware XAI-enabled DT for smart healthcare systems. Additionally, FL is one of the promising solutions that can improve the privacy of healthcare. However, addressing the lack of explainability could be a future research direction. Finally, a real-time function that details the data flow and integration procedure of the proposed XAI-enabled DT framework is intended to be carried out in the future.

## Figures and Tables

**Table 1:** Comparison between this paper and related studies in the recent literature.

Title	XAI’ Security	DT’ Security	Healthcare Systems	Security
[18]	NO	YES	YES	YES
[19]	NO	PARTIAL	YES	PARTIAL
[5]	NO	NO	YES	PARTIAL
[20]	PARTIAL	NO	YES	PARTIAL
[3]	YES	NO	YES	YES
[21]	NO	NO	YES	PARTIAL
[22]	NO	YES	YES	PARTIAL
[23]	NO	NO	NO	PARTIAL
[4]	NO	NO	YES	NO
[24]	NO	NO	YES	NO
[17]	NO	NO	YES	PARTIAL
[25]	NO	NO	YES	PARTIAL
[26]	NO	NO	YES	PARTIAL
[27]	YES	NO	YES	YES
[28]	NO	NO	YES	PARTIAL
This paper	YES	YES	YES	YES

**Table 2:** Summary of Literature.

Title	XAI	DT	Healthcare	Security	Summary
[18]	NA	H	H	L	It proposes an AI-enabled DT healthcare system. It briefly talks about security challenges of DT in healthcare domain
[5]	H	H	H	L	It discusses the importance of XAI and IML to ensure the trustworthiness of AI-enabled DT for healthcare. A detailed explanation of the differences between XAI and IML and a brief discussion about DT’s security were provided.
[20]	H	NA	H	L	It discusses current challenges that face healthcare industry when adopting a metaverse technology including security. Briefly talks about XAI security.
[3]	H	NA	H	H	A comprehensive survey on the potential of using XAI in the security domain of B5G. It discusses some possible adversarial attacks against AI-enabled smart healthcare systems and how XAI can improve the robustness of Healthcare systems.
[21]	H	NA	M	L	A comprehensive survey of AI and XAI methods that are used in the industry 4.0 including smart healthcare. Some XAI tools were discussed, but it does not discuss the security of DT
[22]	NA	H	H	L	It discusses about the importance of improving the robustness of interactive medium between DT and AI. An example of a causative adversarial attack against ML was presented. It discusses about the potential of improving DT’s security by using XAI.
[23]	H	NA	NA	H	Limitations of AI in cybersecurity that necessitate the adaptation of XAI were discussed. However, it does not focus on the security of XAI-enabled DT
[4]	NA	NA	H	NA	A comprehensive survey of AI and XAI methods that are used in the industry 4.0 including smart healthcare. Some XAI tools were discussed, but it does not focus on the security of XAI-enabled DT
[24]	H	NA	H	NA	It addresses some challenges presented in healthcare when using XAI. For example, accuracy versus explainability, human involvement, and explanation assessment.
[17]	M	M	H	H	Security and privacy issues of eHealth were diseased. A brief discussion about XAI-enabled DT in eHealth were presented.
[23]	NA	NA	M	M	It briefly discusses about the benefits and challenges of using FL in smart healthcare systems. However, it does not focus on security of XAI-enabled DT
[26]	L	NA	H	H	Potential benefits of using FL in smart healthcare systems for security improvement were discussed. Specifically, it provides a scenario of using FL to reduce the likelihood of data attribute and inference attacks. A brief discussion about one of the issues of FL is a lack of explainability.
[27]	H	NA	L	L	Briefly discusses the vulnerabilities of XAI in B5G/6G including smart healthcare. Benefits of using FL as a solution for improving the privacy of XAI. XAI security in Healthcare, but not DT.
[28]	NA	NA	H	H	It provides a review of general cybersecurity challenges and solutions in healthcare industry. However, it does not focus on the security of XAI-enabled DT.
This paper	H	H	H	H	A survey of related studies that discuss the security of XAI and DT used in smart healthcare systems. A scenario of potential adversarial attacks against XAI-enabled DT healthcare systems is presented.

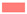
 High Coverage, 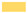 Medium Coverage, 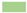 Low Coverage, 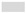 Not Applicable.

**Table 3:** 6W for Designing XAI.

Why?	Who?	What?	Where?	When?	How?
Why is an explanation needed?	Who needs an explanation?	What to explain?	Where the explanations should be made available?	When the explanation should be given?	How the explanation should be generated?
Decision-making	Creators	System predictions	As a prediction remark	Pre-model.	NLP (textual)
Debugging	System operators	Possible vulnerabilities.	As a part of security notification/report	Post-hoc.	Visual (trees or graphs)
Improving Security or accountability	End users	System specifications.	Explanation-as-a-service	In-model	Gamification
Improving performance		Threat model.		After attacks.	

**Table 4:** Common adversarial attacks and defenses.

	Causative Attack	Exploratory Attack
Attack	Poisoning	Probing
	Red Herring	Evasion
	Label-Flipping	Reverse Engineering
		Good Words Attack
Defense	RONI	Randomization
	Game Theory based	Disinformation
	Multiple Learners	

**Table 5:** Evaluation metrics [42].

Metric	Description	Function
Accuracy	The ability of a classifier to correctly find spam/non-spam	$\frac{TP+TN}{TP+FP+TN+FN}$
Recall	The ability of a classifier to correctly find spam	$\frac{TP}{TP+FN}$
Precision	The ability of a classifier to not misclassify spam	$\frac{TP}{TP+Fp}$
F1 Score	The harmonic mean of precision and recall	$\frac{2TP}{2TP+fp+FN}$

**Table 6:** Classification performance of Random Forest.

	Precision	Recall	F1-Score	Support
0	0.96	0.91	0.93	3404
1	0.12	0.24	0.16	173
accuracy			0.88	3577
macro avg	0.54	0.57	0.55	3577
weighted avg	0.92	0.88	0.9	3577

**Table 7:** Classification performance of Logistic Regression.

	Precision	Recall	F1-Score	Support
0	0.97	0.76	0.86	3404
1	0.11	0.6	0.19	173
accuracy			0.72	3577
macro avg	0.54	0.68	0.55	3577
weighted avg	0.93	0.76	0.82	3577

**Table 8:** Classification performance of Support Vector Machines.

	Precision	Recall	F1-Score	Support
0	0.96	0.77	0.86	3404
1	0.09	0.43	0.15	173
accuracy			0.76	3577
macro avg	0.53	0.6	0.5	3577
weighted avg	0.92	0.76	0.82	3577

**Table 9:** LIME Explanation.

Feature	Value
gender	1.00
hypertension	0.00
heart_disease	0.00
age	61.00
avg_glucose_level	202.21
work_type	1.00
bmi	29.88

**Table 10:** ELI5 Explanation.

Weight?	Feature
+1.988	age
+0.197	bmi
+0.149	avg_glucose_level
−0.291	heart_disease
−0.369	hypertension
−0.376	work_type
−0.394	<BIAS>
−0.784	gender

**Table 11:** Classification performance of Random Forest Under A Label-flipping Attack.

	Precision	Recall	F1-Score	Support
0	0.96	0.91	0.93	3387
1	0.15	0.27	0.19	190
accuracy			0.88	3577
macro avg	0.55	0.59	0.56	3577
weighted avg	0.91	0.88	0.89	3577

**Table 12:** Classification performance of Logistic Regression Under A Label-flipping Attack.

	Precision	Recall	F1-Score	Support
0	0.96	0.73	0.83	3387
1	0.1	0.52	0.16	190
accuracy			0.72	3577
macro avg	0.53	0.62	0.5	3577
weighted avg	0.92	0.72	0.79	3577

**Table 13:** Classification performance of Support Vector Machines Under A Label-flipping Attack.

	Precision	Recall	F1-Score	Support
0	0.96	0.75	0.84	3387
1	0.1	0.47	0.16	190
accuracy			0.74	3577
macro avg	0.53	0.61	0.5	3577
weighted avg	0.92	0.74	0.81	3577

**Table 14:** LIME Explanation Under A Label-flipping Attack.

Feature	Value
gender	1.00
hypertension	0.00
heart_disease	0.00
age	61.00
work_type	1.00
avg_glucose_level	202.21
bmi	29.88

**Table 15:** ELI5 Explanation of Under A Label-flipping Attack.

Weight?	Feature
+1.975	age
+1.227	work_type
+0.237	avg_glucose_level
+0.093	bmi
−0.104	<BIAS>
−0.248	heart_disease
−0.362	hypertension
−0.814	gender

## Data Availability

Data is contained within the article.
